# DEVELOPING A HEALTH INEQUALITY/INEQUITY CONCEPTUAL FRAMEWORK TO SUPPORT EVIDENCE SYNTHESIS STUDIES

**DOI:** 10.1093/geroni/igad104.1794

**Published:** 2023-12-21

**Authors:** Tafadzwa Patience Kunonga, Peter Bower, Barbara Hanratty, Dawn Craig

**Affiliations:** Newcastle University, Newcastle upon Tyne, England, United Kingdom; Division of Population Health, Health Services Research & Primary Care, National Institute for Health and Care Research (NIHR) Older People and Frailty Policy Research Unit, Manchester, England, United Kingdom; Newcastle University, Newcastle upon Tyne, England, United Kingdom; National Institute for Health and Care Research (NIHR) Older People and Frailty Policy Research Unit, Newcastle-upon-Tyne, England, United Kingdom

## Abstract

This study aimed to develop a conceptual framework to guide people conducting evidence syntheses, to enable them to visualise links, and intersectionality between various factors that affect health inequality or inequity. Currently, there is no clarity or guidance on how health inequality or inequity should be defined or considered in evidence synthesis, despite recent improvement in reporting standards. In addition, current guides are limited in their ability to provide a roadmap for reviewers that enables them to visualise the links and intersectionality between various factors that affect health inequality or inequity. A series of consensus development workshops were conducted in Spring 2023. Experts in evidence synthesis and health inequality or inequity participated. The workshops were based on methods used previously by researchers developing clinical guidelines. Workshop discussions were centred on (1) important issues/barriers that prevent researchers from having a consensus definition for health inequality or inequity, and (2) how researchers could consider intersectionality of factors affecting health inequality or inequity? Thematic analysis of workshop data and a prioritisation workshop informed the development of a novel conceptual framework. This will be presented and address in particular, ideas around the accumulation of advantage and disadvantage across the life course alongside other dimensions of health and health inequality or inequity.

